# Diffuse Alveolar Hemorrhage With Avelumab Maintenance Therapy

**DOI:** 10.7759/cureus.15805

**Published:** 2021-06-21

**Authors:** Si Li, Nishant Sharma, Daniel Kazmierski, Mohammad Asim Amjad, Yishan Dong, Yichen Wang, Namita Sharma, Srinivasarao Ramakrishna, Pius Ochieng

**Affiliations:** 1 Internal Medicine, The Wright Center for Graduate Medical Education, Scranton, USA; 2 Internal Medicine, Rochester Regional Health, Rochester General Hospital, Rochester, USA; 3 Mercy Internal Medicine Service, Trinity Health of New England, Springfield, USA; 4 Hematology and Oncology, Geisinger Commonwealth School of Medicine, Scranton, USA; 5 Pulmonary and Critical Care Medicine, Geisinger Commonwealth School of Medicine, Scranton, USA

**Keywords:** immune check-point inhibitor, diffuse alveolar hemorrage, urothelial malignancy, acute hypoxemic respiratory failure, ground-glass opacity

## Abstract

Immune checkpoint blockade is a rapidly expanding therapeutic modality in oncology. However, its adverse effects extend beyond the cytotoxicity of conventional chemotherapy. Pneumotoxicity associated with immune checkpoint therapy presents a diagnostic conundrum that has been further complicated by the COVID-19 pandemic. We report a case of a patient with metastatic urothelial carcinoma who developed diffuse alveolar hemorrhage (DAH) following treatment with avelumab.

## Introduction

Immune checkpoint inhibitors have shown significant survival benefits in cancer patients and have revolutionized the outcomes of various malignancies having a better safety profile compared to chemotherapy [1, 2]. However, their adverse effects range from mild self-limiting to severe life-threatening side effects, which could potentially limit the use of these medications. Avelumab is a human IgG1 monoclonal antibody that targets programmed cell death ligand 1 (PD-L1) of the intrinsic down regulators of immunity and that induces durable remissions in different tumor types [3]. First-line maintenance avelumab therapy in patients with advanced urothelial carcinoma was found to be associated with significantly prolonged overall survival [4, 5]. While increasing the activity of the immune system, such blockade can cause inflammatory side effects. The GI tract, liver, and skin are more commonly affected. Pulmonary, the central venous system, and cardiovascular involvement are also seen [6, 7]. A safety profile evaluation that analyzed 1,738 patients showed that patients receiving avelumab predominantly developed chills, pyrexia, and flushing, which may reflect the human Fc region-induced activation of innate immunity [8]. In an open-label phase 3 trial, 29.4% of patients had immune-related adverse events among the avelumab-treated patients, including 7% of patients with a grade 3 event. The most frequent category was thyroid disorders (12.2%) [4].

## Case presentation

A 69-year-old Caucasian male presents with recurrent high-grade urothelial carcinoma with liver and osseous metastases. Four years ago, he was initially diagnosed with bladder carcinoma in situ and underwent transurethral resection followed by intravesical Bacillus Calmette-Guérin (BCG) therapy, leading to remission. However, one year ago, he had hematuria and underwent cystoscopy, which showed an abnormal lesion of the trigone and bladder neck. Bladder washing suggested high-grade urothelial carcinoma. A subsequent transurethral resection revealed muscle-invasive high-grade urothelial carcinoma. He was also found to have liver and widespread osseous metastases. The patient was started on chemotherapy with cisplatin and gemcitabine and achieved stabilization of disease after four cycles. He was switched to maintenance avelumab for seven months (four months after the diagnosis of metastases). His other past medical history included: cardiac arrest due to ischemic cardiomyopathy and status post coronary artery bypass surgery, implantable cardioverter-defibrillator placement, and paroxysmal atrial fibrillation (diagnosed nine years ago, treated with rivaroxaban since then). He had a history of smoking for more than forty packs/year, quit for eight years, worked as a television producer, and occasionally played hockey games.

**Figure 1:**
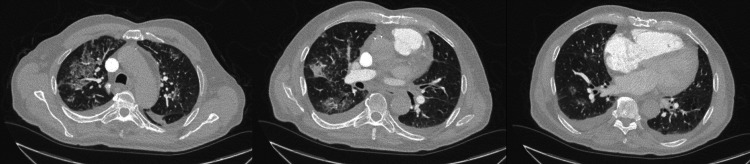
Diffuse bilateral ground glass and more consolidative airspace opacification and small bilateral pleural effusions.

**Figure 2:**
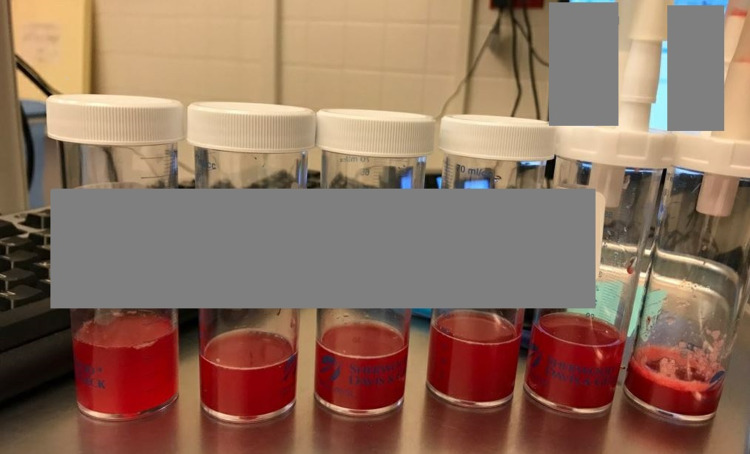
Flexible bronchoscopy revealed progressively more hemorrhagic lavage fluid.

This time, he presented with one month of worsening shortness of breath and weakness. He was hypoxic, with oxygen saturation of 67% upon arrival at his oncology appointment. He was afebrile, alert, had scattered rhonchi and wheezes on lung auscultation. Laboratory results (Table 1) revealed mild leukocytosis. He had an elevated brain natriuretic peptide (BNP), C-reactive protein (CRP), and lactate dehydrogenase (LD). Hemoglobin and platelet counts were within the normal limit. Blood cultures and respiratory pathogen panel including SARS-COV2 polymerase chain reaction (PCR) were negative, c-antineutrophil cytoplasmic antibodies (ANCA) and p-ANCA were also negative. CT chest with intravenous contrast showed new diffuse bilateral ground glass and more consolidative airspace opacification and small bilateral pleural effusions (Figure 1). Transthoracic echocardiogram showed normal ejection function and no significant valvopathy. The patient’s oxygen requirements increased on the second day of hospitalization, and he was transiently started on a high-flow nasal cannula with a 50% fraction of inspired oxygen FiO2 at 50 liters per minute (LPM). Flexible bronchoscopy revealed progressively more hemorrhagic lavage fluid (Figure 2) and bleeding from the right upper lobe. Transbronchial lung biopsies were performed, and samples were sent for cytology and culture. A cytology report from bronchoalveolar lavage (BAL) revealed respiratory epithelial cells, pulmonary macrophages, and mixed inflammatory cells and negative for malignant cells. Cytology of right upper lobe core biopsy was positive for CK7 immunostaining. Uroplakin II immunostaining was focally positive. P40, CK20, and p63 immunostains were negative. GATA-3 immunostain was equivocal. Given the limited tumor cells in the biopsy, the primary source of the tumor could not be identified. Although the urothelial origin of the tumor was favored, rivaroxaban was discontinued. The patient was started on methylprednisolone 250 mg daily for three days after the bronchoscopy, then switched to 60 mg daily. The avelumab was not restarted since this hospitalization. However, the patient died eight days after admission due to respiratory failure despite all treatment attempts.

**Table 1:** Clinical laboratory results.

Variable	Reference Range	On Admission
White cell count (K/uL)	4.00-10.80	11.45
Absolute neutrophils (K/uL)	1.80-7.70	9.13
Hemoglobin (g/dL)	14.0-16.8	13.4
Hematocrit (%)	40.0-48.4	40.2
Platelet count (K/uL)	140-400	301
Brain natriuretic peptide (pg/mL)	<300	3,603
C-reactive protein (mg/L)	<=5	99
Lactate dehydrogenase (U/L)	<=250	418
Alkaline phosphatase (U/L)	35-130	226

## Discussion

Our patient on maintenance avelumab for metastatic urothelial carcinoma presented with subacute dyspnea attributed to diffuse alveolar hemorrhage (DAH). DAH, as an uncommon type of lung injury, is rarely documented in patients receiving checkpoint inhibitor therapy. We hypothesize that DAH is related to the use of avelumab, which may potentially increase the risk of hemorrhage in metastatic cancer. To our knowledge, this is the first case of avelumab-induced DAH in patients with metastatic urothelial carcinoma reported in the literature.

To date, DAH caused by immune checkpoint inhibitors has been reported in a few case reports and case series. Shannon et al. presented a case of fatal DAH following nivolumab therapy [9]. Takahashi et al. described the development of Goodpasture’s disease after eight cycles of nivolumab treatment with hemoptysis symptoms and diffuse ground-glass opacities on the CT scan. The patient died despite methylprednisolone pulse therapy and plasma exchange [10]. In another case of nivolumab-induced DAH, with three days of methylprednisolone pulse administration (1 g/day) followed by prednisolone therapy (40 mg/day), the patient demonstrated clinical improvement with the disappearance of ground-glass opacities [11]. Sugano et al. highlighted the relationship between interstitial lung disease with alveolar hemorrhage and pembrolizumab treatment in a patient without respiratory symptoms [12].

Patients with DAH can present with hemoptysis and dyspnea, in our case, it developed after seven months of avelumab maintenance treatment. However, hemoptysis may be absent in one-third of patients at presentation [13]. This raises the concern of underdiagnosed PD-L1 inhibitor-induced DAH due to nonspecific symptomatology in our patient and immune checkpoint inhibitors associated DAH reported by others. Characteristic radiographic findings include diffuse ground glass or consolidative opacities and tend to be more central than peripheral [14]. The diagnosis of DAH is supported by findings of progressively more hemorrhagic lavage returns during sequential BAL and the presence of hemosiderin-laden macrophages on cytologic staining [15]. Other serum findings, such as leukocytosis, the elevation of erythrocyte sedimentation rate, and CRP, can also be seen but not specific.

The underlying pathophysiology of avelumab-induced DAH is still unknown. However, the potential mechanisms may include pneumonitis caused by dysregulated immunologic homeostasis, increasing T-cell activity, increasing levels of preexisting autoantibodies and inflammatory cytokines, and enhancement of complement-mediated inflammation [6, 16]. The subsequent disruption of the alveolar-capillary basement membrane caused bleeding into the alveolar spaces in the setting of long-term anticoagulation therapy. Rivaroxaban-associated alveolar hemorrhage is also rare, and no definitive evidence of a direct causative relationship has been established [17]. In our case, rivaroxaban was started nine years ago, which is unlikely to cause the recent hemorrhage. Kanaoka et al. described bland pulmonary hemorrhage as a histopathological feature in a case of durvalumab-induced DAH. The author also argues for CD4 and CD8 immunostaining as essential in distinguishing lung injury as an immune-related adverse event from other types of lung injuries [18].

The relationship between immune checkpoint inhibitors and the incidence of treatment-related DAH has not been well studied. In addition to causative drug discontinuation and airway supportive care, high-dose methylprednisolone therapy may have a critical role if the alveolar hemorrhage is suspected to be immune-mediated [19]. Previous studies also suggested responses can be elicited by the steroids in immune checkpoint inhibitors induced DAH [11, 12]. The treatment should not be delayed while pending etiology evaluation due to the high mortality. Intermittent intravenous pulse dose can be considered to minimize treatment-related complications, including bone marrow suppression and opportunistic infections.

## Conclusions

Avelumab-induced DAH remains a diagnostic challenge that is based on excluding other potential causes in a usually complex clinical context. Early recognition and discontinuation of the offending medication are crucial. Our case reinforces the importance of being vigilant with patients on avelumab who present with progressive dyspnea and suspicion of DAH, as early recognition and treatment can be lifesaving. DAH should also be suspected in patients receiving avelumab with ground glass or consolidative lesions on imaging studies. Prompt bronchoscopy and sequential BAL can expedite the evaluation, even in the absence of hemoptysis and decreasing hemoglobin level. The multidisciplinary discussion also serves as a key part of the diagnostic decision-making process given the wide range of potential organ system involvement. Future evaluation regarding management, monitoring, and preventive strategies is warranted.
